# Chronic Facial Abscess Mimicking Cervicofacial Actinomyces From Dermal Filler Migration: Case Report

**DOI:** 10.2196/80278

**Published:** 2026-02-03

**Authors:** Monika Ziogaite, Sarah Mannlein, Nicole Bender, Scott J Mahlberg

**Affiliations:** 1 College of Osteopathic Medicine, Kansas City University, 1750 Independence Ave, Kansas City, MO, 64106, United States, 1 816-654-7000; 2 Colorado Center for Dermatology & Skin Surgery, Longmont, CO, United States; 3 Sagis Dermatopathology, Houston, TX, United States

**Keywords:** dermal filler, foreign body reaction, facial abscess, granulomatous inflammation, filler complications, cosmetic dermatology

## Abstract

Dermal fillers are commonly used for facial augmentation, but delayed complications such as granulomatous inflammation and filler migration can mimic chronic bacterial infections, such as cervicofacial actinomycosis, and lead to diagnostic misdirection. We present the case of a woman aged 56 years with a chronic, draining abscess on the right cheek that persisted for 3 years and was initially suspected to represent cervicofacial actinomycosis. Tissue cultures were negative, and histopathologic analysis following excisional biopsy revealed polymethyl methacrylate microspheres and hyaluronic acid surrounded by granulomatous inflammation and reactive lymphoid aggregates, consistent with a foreign body reaction to dermal filler. The patient experienced complete resolution after surgical excision. This case underscores the diagnostic challenges posed by delayed filler complications and highlights the importance of considering prior cosmetic procedures in patients with chronic facial abscesses.

## Introduction

The use of dermal fillers for facial augmentation has increased significantly, with both temporary (hyaluronic acid) and semipermanent or permanent fillers (polymethyl methacrylate [PMMA], calcium hydroxylapatite, silicone) [1]. While most complications occur immediately or within weeks, delayed reactions, including granuloma formation and filler migration, can present months to years after injection [2].

Foreign body granulomas are a known complication of PMMA-based fillers (Bellafill/Artefill; Suneva Medical Inc) and result from a chronic inflammatory response to nondegradable microspheres [3]. These reactions may be triggered by delayed hypersensitivity, biofilm formation, or immune dysregulation and can resemble infectious or inflammatory processes. They can also closely mimic chronic infectious processes such as cervicofacial actinomycosis, characterized by draining sinuses and subcutaneous abscesses, often prompting an extensive infectious workup before the true etiology is recognized [4]. Diagnosis relies on histopathologic evaluation, which typically reveals multinucleated giant cells, lymphoid aggregates, and fibrosis surrounding filler particles [3,4].

This case highlights the importance of early recognition of iatrogenic causes in the differential diagnosis of chronic facial abscesses and underscores the long-term risks associated with semipermanent fillers, particularly PMMA-based products. Given the varied histopathologic presentations of different filler materials, distinguishing PMMA from other injectables is crucial for accurate diagnosis and management.

## Ethical Considerations

The authors obtained written consent from the patient for their photographs and medical information to be published in print and online, with the understanding that this information may be publicly available. Patient consent forms were not provided to the journal but are retained by the authors.

## Case Report

A woman aged 56 years presented with a chronic, nonhealing, draining abscess localized to the right cheek. Characterized by intermittent drainage, localized tenderness, and surrounding erythema, the nodule persisted for approximately 3 years, during which time the patient sought care from specialists on at least one occasion. The patient denied systemic symptoms such as fever, chills, dental caries, oral drainage, pain with salivation, or malaise. Past medical history was noncontributory, and the patient had no known history of immunosuppression, diabetes, or recurrent skin infections.

**Figure 1.**
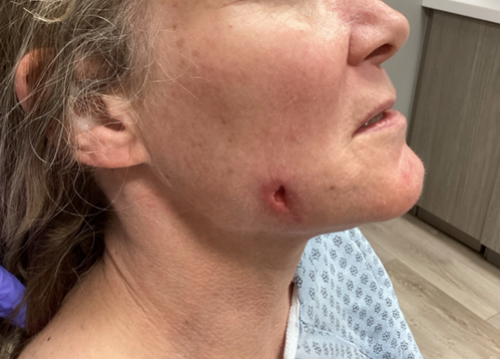
Nonhealing ulcer with surrounding erythema and induration on the right cheek.

On physical examination, the deep nodule was ulcerated, with erythematous borders localized to the right inferior central malar cheek. The ulcer base exhibited crusting and purulent material, with 3 cm of surrounding induration (Figure 1). No regional lymphadenopathy was present.

**Figure 2.**
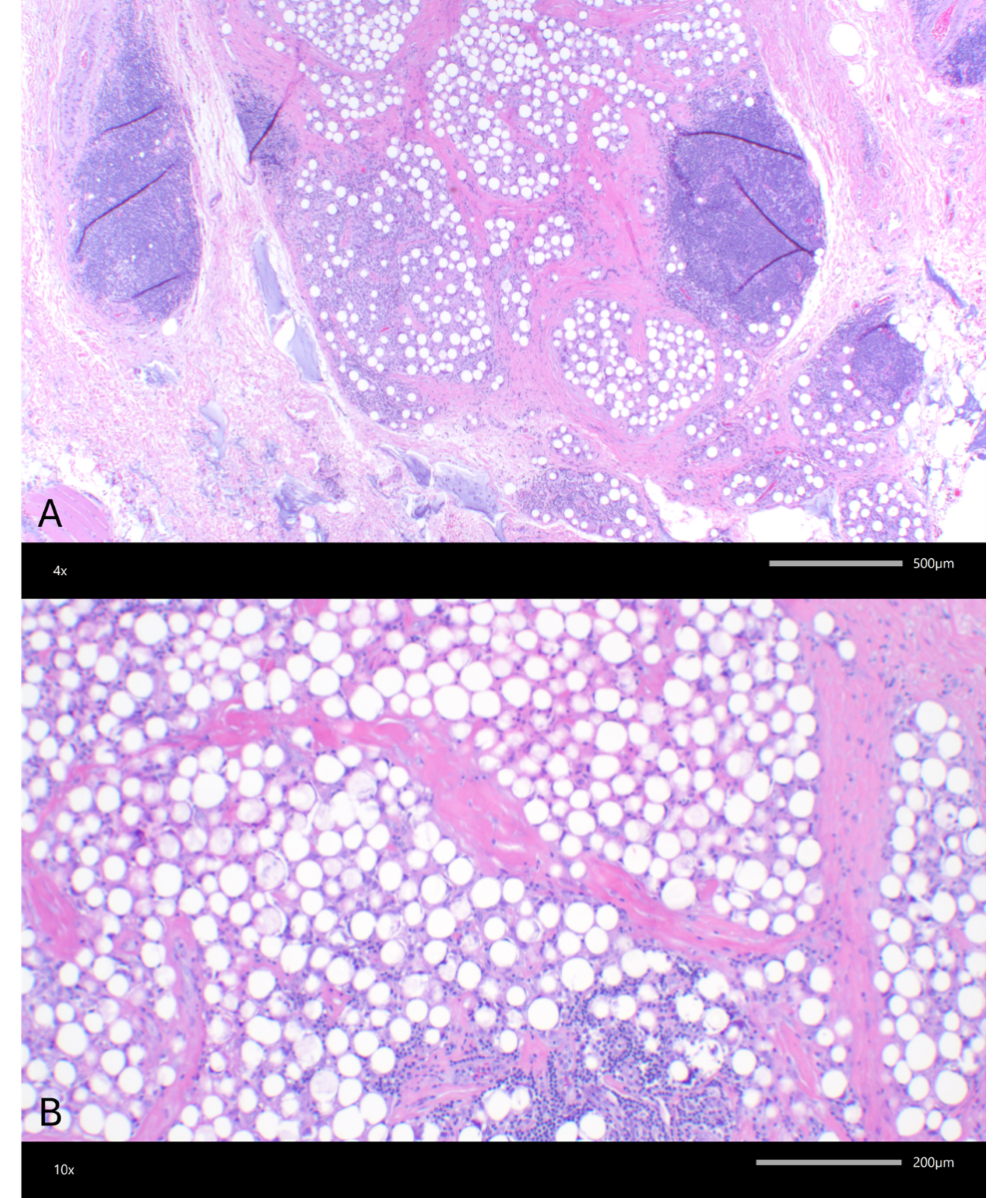
Histologic features consistent with a foreign body reaction to polymethylmethacrylate (PMMA) filler. (A) Incisional biopsy with numerous rounded vacuolated spaces, 30‐50 μm in size, consistent with PMMA spherules and surrounding granulomatous inflammation and reactive lymphoid aggregates. Adjacent homogeneous basophilic material consistent with hyaluronic acid is also present (hematoxylin and eosin stain; 4x). (B) Higher magnification of PMMA spherules (hematoxylin and eosin stain; 10×).

Given the persistent nature of the abscess, a tissue culture was obtained via punch biopsy, which was negative for bacterial, fungal, and atypical mycobacterial growth. Due to the size and depth of the lesion, an excisional biopsy was performed to identify potential inflammatory or neoplastic pathology. Histopathologic analysis (Figure 2) revealed foreign body granulomas with abundant reactive lymphoid tissue, along with an accumulation of PMMA microspheres and hyaluronic acid, consistent with semipermanent dermal fillers. Surrounding granulomatous inflammation was also noted. Special stains, including Grocott methenamine silver, Fite, and Gram stains, were negative for fungi, mycobacteria, and bacteria, ruling out infectious etiologies.

**Figure 3.**
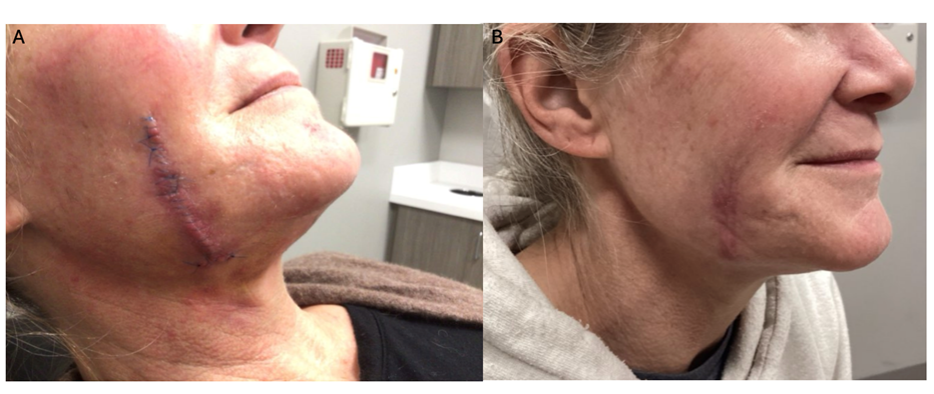
(A) Postoperative appearance at 1 week showing early healing. (B) Postoperative appearance at 4 weeks with complete resolution and no recurrence.

The patient was seen for suture removal at 1 week (Figure 3) with an uneventful postoperative course. At 4 weeks postoperatively, the surgical site was well healed, with no signs of infection or recurrence (Figure 3). Although initially unable to recall prior dermal filler use, review of the pathology report prompted the patient to remember having received Bellafill (Suneva Medical Inc) approximately 6 years prior and Restylane (Galderma Laboratories) approximately 10 years earlier, both for treatment of acne scarring on the cheeks.

## Discussion

While dermal fillers are widely used for aesthetic enhancement, their delayed complications remain an evolving area of clinical concern. Although most adverse reactions occur shortly after injection, late-onset complications can develop months to years later, often leading to diagnostic uncertainty [5]. Unlike temporary hyaluronic acid–based fillers, PMMA and other nondegradable materials persist within tissues long-term, increasing the risk of prolonged inflammatory responses [6]. The delayed presentation of PMMA-related granulomas frequently results in misdiagnosis as infection or inflammatory dermatoses, delaying appropriate intervention [7].

While complications such as filler migration and granulomatous reactions are well documented, the development of a chronic filler reaction mimicking a cervicofacial actinomycetoma is rare. Actinomycetomas are chronic, subcutaneous infections caused by filamentous bacteria, characterized by abscesses, draining sinuses, and granule production [8]. The striking clinical resemblance between the foreign body granuloma in this case and a deep-seated actinomycotic infection underscores the diagnostic challenges posed by delayed filler reactions. This case highlights the need for broad infectious and histopathologic workups in atypical, chronic soft tissue infections to prevent unnecessary antibiotic treatment and delayed surgical intervention.

**Figure 4.**
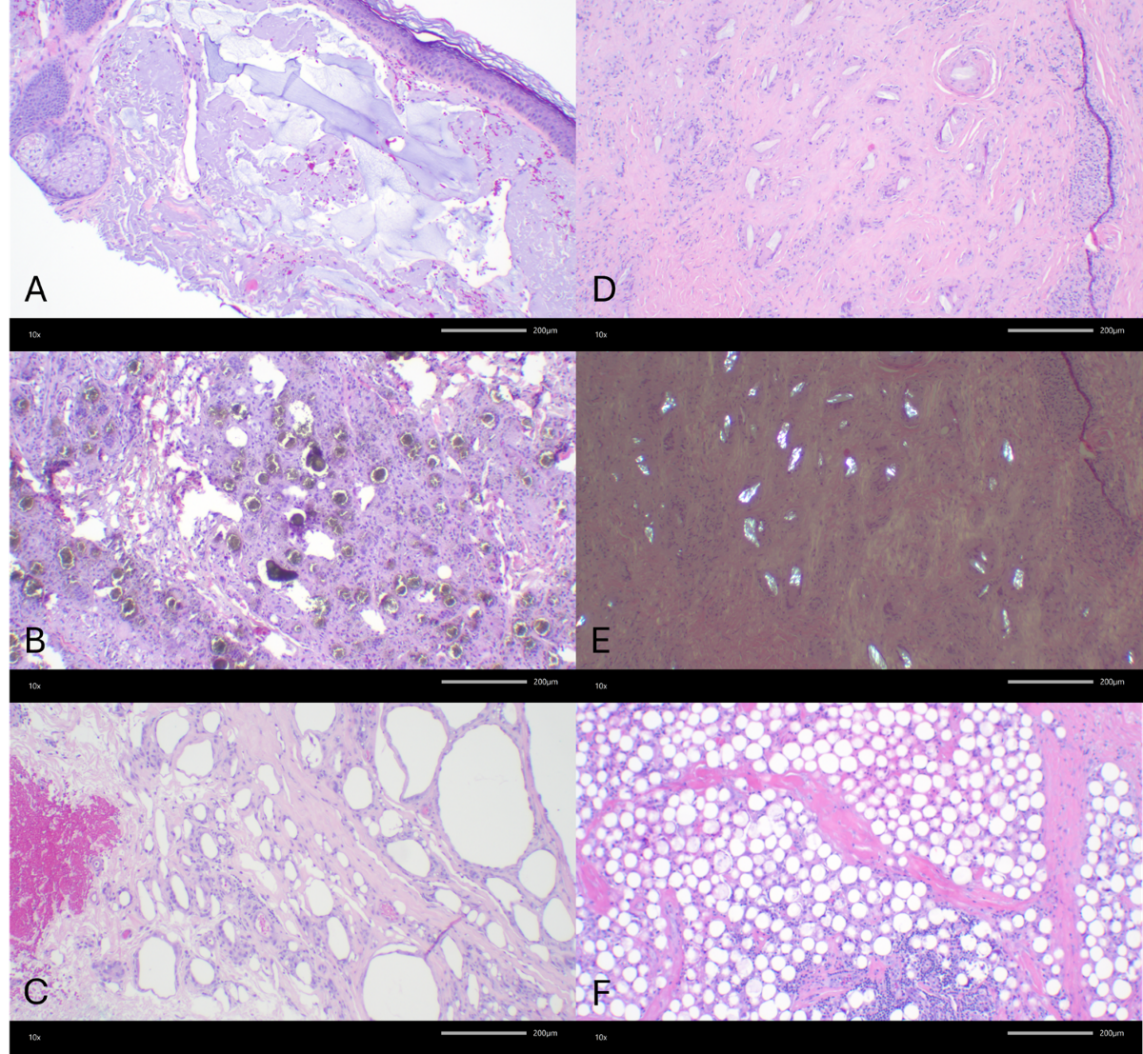
Histologic features consistent with foreign body reactions to soft tissue augmentation materials. (A) Pools of wispy homogeneous basophilic material consistent with hyaluronic acid (hematoxylin and eosin; 10×). (B) Gray-green granular nonrefractile microspheres consistent with calcium hydroxylapatite with surrounding granulomatous inflammation (hematoxylin and eosin; 10×). (C) Variably sized empty lipidlike vacuoles within histiocytes consistent with silicone granuloma (hematoxylin and eosin; 10×). (D) Oval, rhomboidal, and rice-shaped clear refractile and polarizable structures consistent with poly-L-lactic acid (PLLA) particles within histiocytes (hematoxylin and eosin; 10×). (E) Polarization of PLLA fragments (hematoxylin and eosin; 10×). (F) Fairly uniform 30‐50-µm rounded vacuolated spaces consistent with polymethylmethacrylate spherules (hematoxylin and eosin; 10×).

Histopathologic evaluation is essential for diagnosing PMMA-related granulomas, which are characterized by multinucleated giant cells, chronic lymphohistiocytic infiltrates, and fibrosis surrounding filler particles [9]. In this case, a negative infectious workup and biopsy findings of PMMA microspheres with reactive lymphoid tissue confirmed the diagnosis and guided treatment. Management remains challenging, as PMMA-based fillers lack a reversal agent comparable to that of hyaluronic acid fillers [10]. While intralesional corticosteroid injections may offer partial improvement, surgical excision is often required for definitive diagnosis and treatment, as in this case [4]. Given the histologic variability among filler types, distinguishing PMMA granulomas from reactions to calcium hydroxylapatite, poly-L-lactic acid, and silicone is critical for guiding optimal management strategies (Figure 4).

While PMMA fillers are used less frequently than hyaluronic acid–based products, their potential for chronic inflammatory complications requires heightened clinical awareness and a detailed risk-benefit discussion prior to injection. Semipermanent fillers pose unique challenges due to their prolonged tissue retention and risk of delayed reactions. Clinicians should maintain a high index of suspicion for foreign body granulomas and probe for a history of prior filler use in cases of chronic, nonhealing facial abscesses, particularly when standard antimicrobial therapy fails or imaging reveals localized nodularity. Following excision, patients should be informed of and monitored for delayed recurrence as well as contralateral lesions, as these may occur months to years later. This case highlights the value of histopathologic microbiologic evaluation in diagnosing facial abscesses, the limitations of nonsurgical management for PMMA-induced granulomas, and the need for increased awareness of iatrogenic factors in chronic soft tissue reactions.

## Abbreviations

PMMA: polymethyl methacrylate
